# Supplementary figures and images for: Burkholderia genome mining for nonribosomal peptide synthetases reveals a great potential for novel siderophores and lipopeptides synthesis
Source: Microbiologyopen. 2016 Apr 5;5(3):512–26. doi: 10.1002/mbo3.347 (PMC4906002; doi:10.1002/mbo3.347)

Figure S1

A

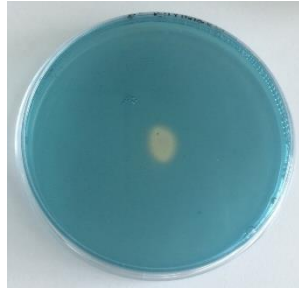

*B. phymatum* STM85

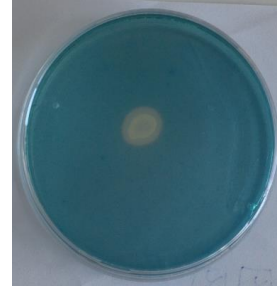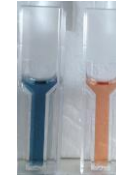

### *B. ambifaria* AMMD

B

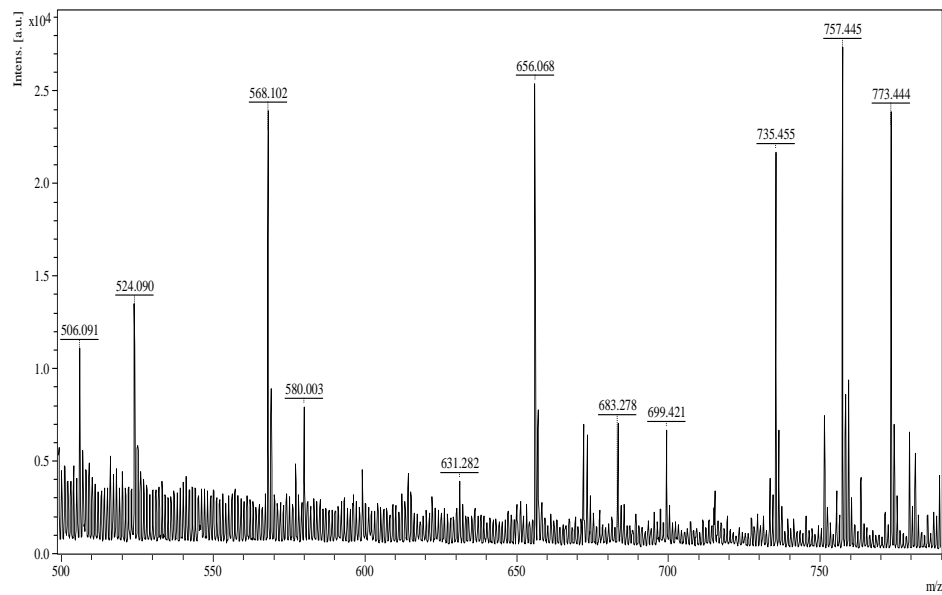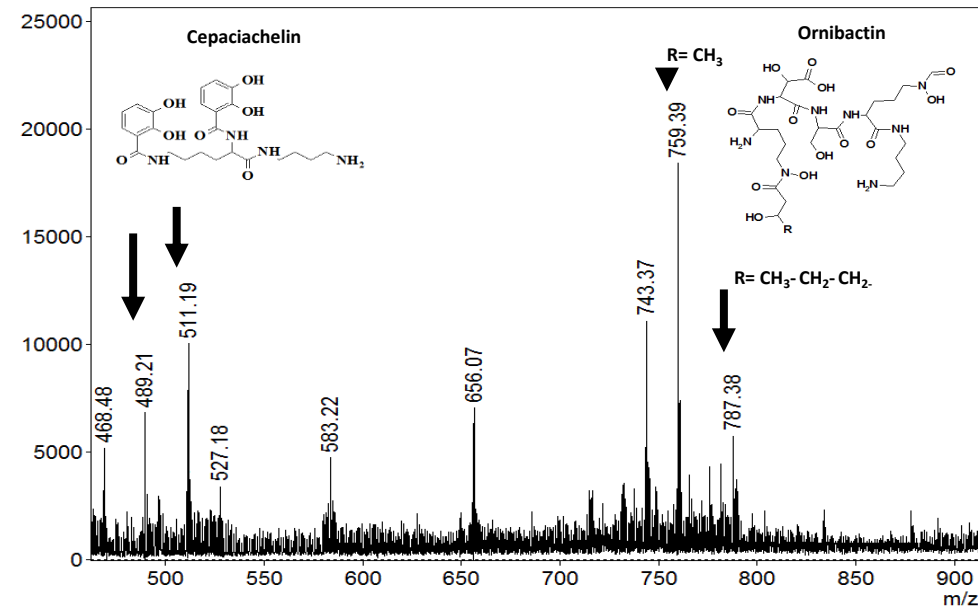

Supplement: Supplementary file 1 — Figure S1. Detection of siderophores by CAS assays and MALDI‐ToF. Left: Burkholderia phymatum STM85, right: Burkholderia ambifaria AMMD. (A) CAS assays. The color change from blue to orange indicates the presence of iron‐chelating compounds. (B) MALDI‐ToF spectra. Peaks corresponding to cepaciachelin and ornibactin are pointed out by arrows. [file MBO3-5-512-s001.pdf]

Figure S2

A

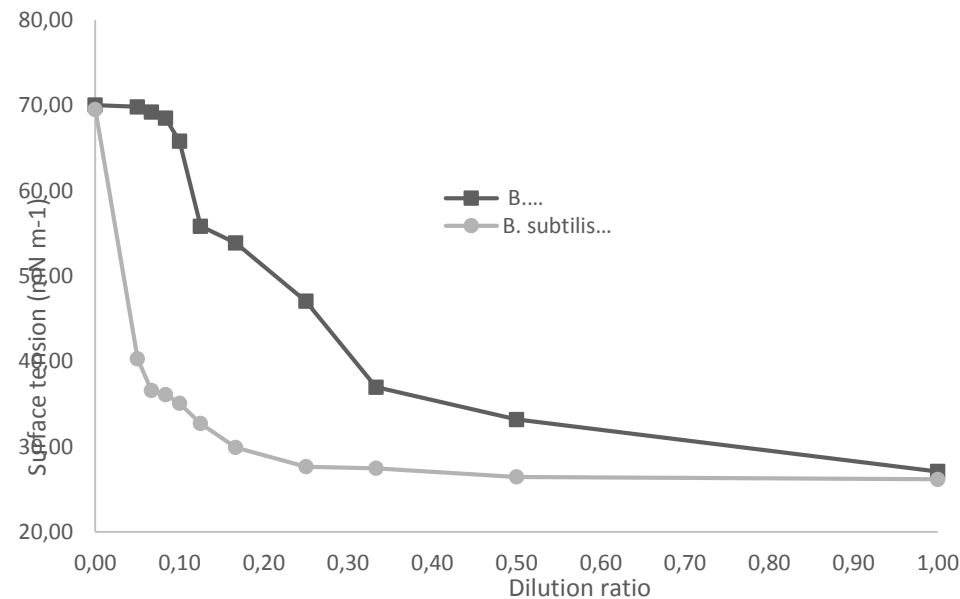

B

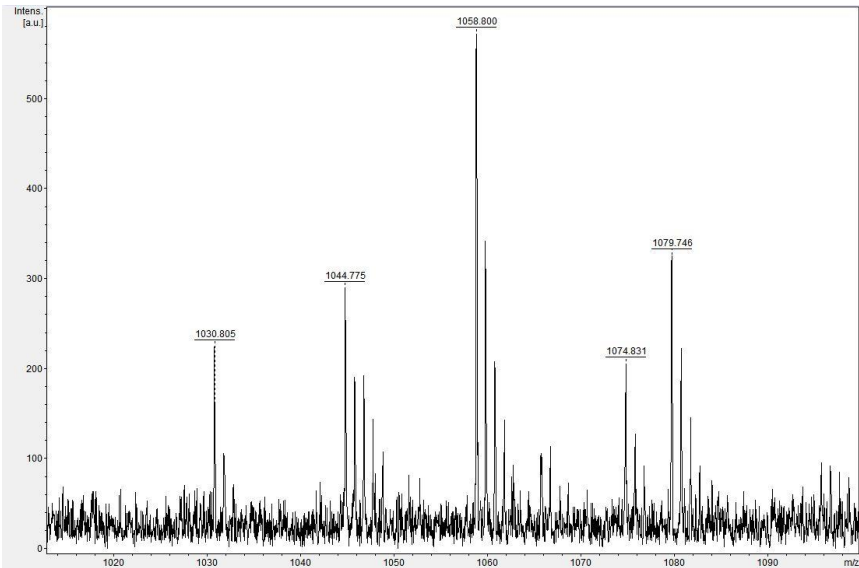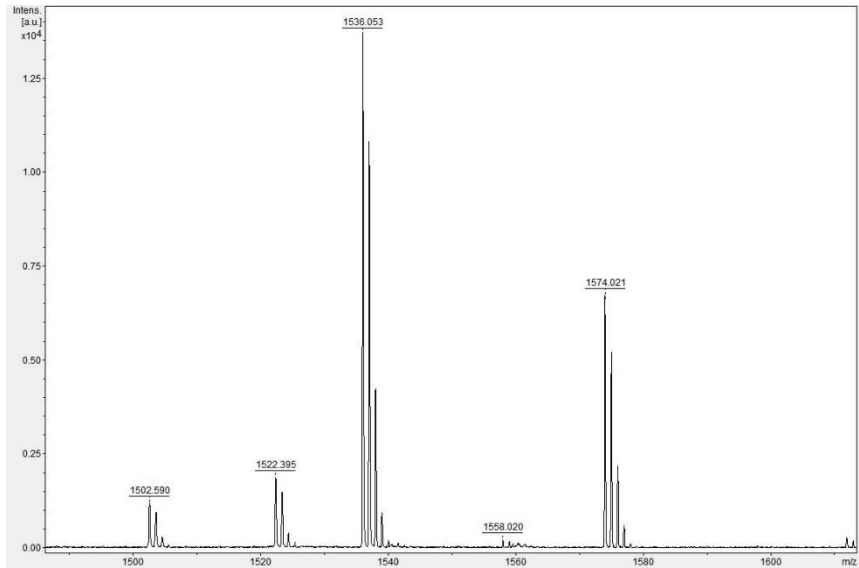

Supplement: Supplementary file 2 — Figure S2. Detection of lipopeptides produced by Burkholderia rhizoxinica HKI 454. The strain was grown in Landy medium. (A) Surface tension, (B) MALDI‐ToF. A group of peaks were observed, characteristic of lipopeptide detection with different fatty acid chain lengths, with mass differences of 14 Da (1522 and 1536). [file MBO3-5-512-s002.pdf]

Figure S3

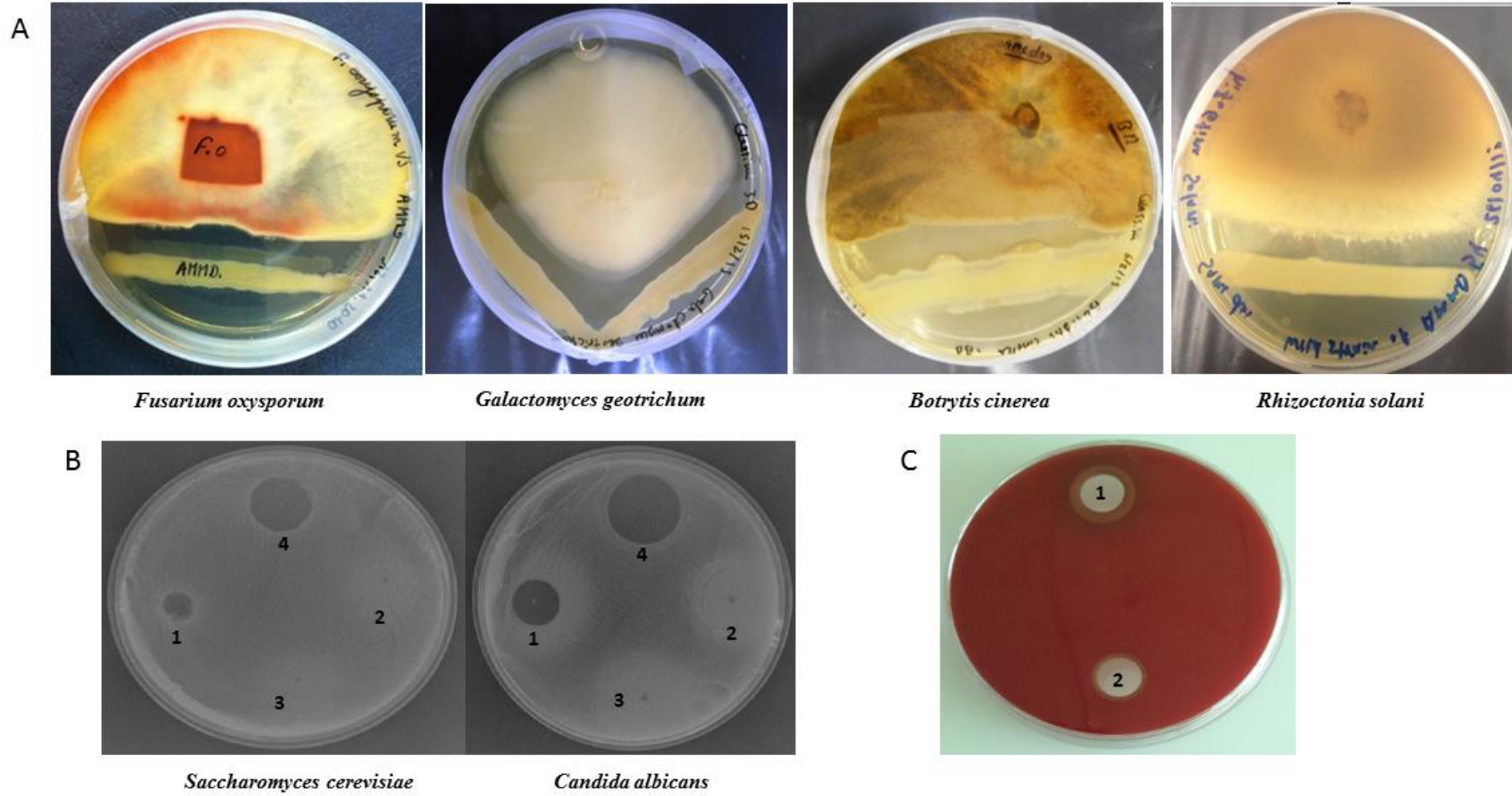

Supplement: Supplementary file 3 — Figure S3. Biological activities of Burkholderia ambifaria AMMD. (A) antifungal activity. Burkholderia ambifaria AMMD was inoculated as a line in front of the fungal target on PDA medium. The fungi are mentioned under the plates. Plates were photographed 5 days after inoculation. (B) Antiyeast activity. The yeasts are mentioned under the plates. 1: AMMD wild type, 2: ΔBamb_6472 mutant, 3: sterile medium, 4: ethanol 70%. (C) Hemolytic activity on horse bood (5%) LB medium, 50 μL of supernatant obtained after 48 h of growth were used to fill the wells. 1: AMMD wild type, 2: ΔBamb_6472 mutant. [file MBO3-5-512-s003.pdf]
