# Supplementary material for: Burkholderia genome mining for nonribosomal peptide synthetases reveals a great potential for novel siderophores and lipopeptides synthesis
Source: Microbiologyopen. 2016 Apr 5;5(3):512–26. doi: 10.1002/mbo3.347 (PMC4906002; doi:10.1002/mbo3.347)
Supplement: Supplementary file 4 — Table S1. Strains, plasmids, and primers used in this study. [file MBO3-5-512-s004.pdf]

Table S1

**Strains, plasmids, and primers**	**Description**	**Reference or source**
**Strains**		
B. ambifaria AMMD LMG 19182	Wild type; Gm^s	LMG
B.ambifaria AMMD-Δbamb_6472	Mutant with deletion of NRPS gene bamb_6472	This study
B. phymatum STM815		DSMZ 17167
B. rhizoxinica HKI 454		DSMZ 19002
Escherichia coli WM3064	Strain for conjugation; λ pir, DAP auxotroph	Saltikov and Newman, 2003
Escherichia coli DH5α	Host of cloning	ProBioGEM lab stock
Listeria innocua 51742	Target for antibacterial activity	ATCC
Micrococcus luteus	Target for antibacterial activity	ProBioGEM lab stock
Candida albicans ATCC10231	Target yeast strain for fungicide testing	ProBioGEM lab stock
Saccharomyces cerevisiae	Wild type	ProBioGEM lab stock
Saccharomyces cerevisiae InvSc1	Yeast strain for *in vivo* recombination	Invitrogen
	MAT a/*MAT* α *leu* 2/*leu* 2 *trp* 1-289/*trp* 1-289 *ura* 3-52/*ura* 3-52 *his* 3-Δ1/*his* 3-Δ1	
Botrytis cinerea R16	Phytopathogen	ProBioGEM lab stock
Fusarium oxysporum	Phytopathogen	ProBioGEM lab stock
Galactomyces geotrichum MUCL2859	Phytopathogen	ProBioGEM lab stock
Rhizoctonia solani S010-1	Phytopathogen	ProBioGEM lab stock
**Plasmids**		
pMQ30	7.6 kb mobilizable suicide vector used for gene replacement in *pseudomonas* : SacB, URA3, Gm^r	Shanks *et al* ., 2006
pMQ30Δ6472	pMQ30 containing two fragments of 1kb of the NRPS biosynthesis gene bamb_6472	This study
**Primers (5’-----> 3’)**		
Up6472-F	*GGAATTGTGAGCGGATAACAATTTCACACAGGAAACAGCTG* CGATCCAGTACCGCGACTAC	This study
Up6472-R	*TCGGAAGGGAATAGGTCAGC* TGATCGGTGACCAGTACGTT	This study
Down6472-F	*AACGTACTGGTCACCGATCA* GCTGACCTATTCCCTTCCGA	This study
Down6472-R	*CCAGGCAAATTCTGTTTTATCAGACCGCTTCTGCGTTCTGAT* TCCTTTTGCACAGGTTGACG	This study
