# Supplementary material for: Burkholderia genome mining for nonribosomal peptide synthetases reveals a great potential for novel siderophores and lipopeptides synthesis
Source: Microbiologyopen. 2016 Apr 5;5(3):512–26. doi: 10.1002/mbo3.347 (PMC4906002; doi:10.1002/mbo3.347)
Supplement: Supplementary file 5 — Table S2. Ornibactin, malleobactin, and phymabactin gene clusters. “+” and “−” mention the relative orientation of the genes. Numbers represent the size of the proteins (in AA). Colors represent the role of the protein in the siderophore production: green for NRPS biosynthesis, orange for accessory enzymes, blue for uptake, red for regulation, and yellow for unknown function. [file MBO3-5-512-s005.pdf]

Table S2

siderophore	strain	Extracytoplasmic σ70 (regulation)	mbtH	SyrP like	ABC transporter	transporter permease	ferric iron reductase	periplasmic binding protein	Hypothetical Protein	cyclic peptide transporter	NRPS	NRPS	Hypothetical Protein	Ornithin monooxygenase	TonB dpdt receptor	Folate formyltransferase	ornibactin biosynthesis
		mba F	*mba* G	*mba* H	*mba* I	*mba* J	*mba* K	*mba* L	*mba* M	*mba* N	*mba* A	*mba* B		*mba* C	*mba* D	*mba* E	
malleobactin	mallei ATCC 23344	+207	+80	+338	+296	+706	+268	+342	-255	-562	0	+1732	0	+468	+741	?	0
malleobactin	mallei NCTC 10229	+237	+80	+333	+296	+706	+268	+398	-255	-562	+3294	+1732	0	+517	+752	?	0
malleobactin	mallei NCTC 10247	+237	+80	+333	+296	+706	+268	+398	-255	-562	+3294	+1732	0	+468	+736	?	0
malleobactin	mallei SAVP1	+237	+80	+338	+296	+706	+268	+398	-191	-562	+3297	+1732	0	+468	+736	?	0
malleobactin	phytofirmans PsJN	+223	+79	+344	+288	+702	+272	+342	-95	-588	+3230	+1674	0	+456	+745	+280	0
malleobactin	pseudomallei 1026b	+257	+80	+338	+296	+706	+264	+342	-191	-581	+3287	+1732	0	+468	+737	+279	0
malleobactin	pseudomallei 1106a	+237	+80	+333	+296	+706	+268	+398	+250	-562	+3290	+1741	0	+468	+753	+279	0
malleobactin	pseudomallei 1106B	+237	+80	+338	+296	+706	+264	+385	-191(+123)	-562	+3290	+1741	0	+468	+753	?	0
malleobactin	pseudomallei 1710a	+237	+82	+338	+296	+706	+264	+342	-220(+59)	-562	+1480+1908	+1739	0	+458	+753	?	0
malleobactin	pseudomallei 1710b	+237	+82	+ 338	+ 296	+ 706	+ 268	+429	- 267	-562	+3287(+3293)	+1739(+1772)	0	+495	+737	+279	0
malleobactin	pseudomallei 668	+237	+82	+338	+296	+706	+264	+398	-253	-562	+3290	+1745	0	+468 (MbaA)	+753	+279	0
malleobactin	pseudomallei BPC006	+237	+80	+338	+296	+706	+268	+398	-191(+129)	-581	+3291	+1732	0	+517	+737	?	0
malleobactin	pseudomallei K96243	+207	+80	+338	+296	+706	+268	+382	-191	-581	+3290	+1748	0	+468	+753	+279	0
malleobactin	pseudomallei MSHR146	+237	+80	+336	+296	+706	+268	+398	-191	-562	+3288	0	0	+468	+737	+279	0
malleobactin	pseudomallei MSHR305	+237	+80	+340	+296	+706	+268	+398	-191	-576	+3292	+1736	0	+468	+737	+279	0
malleobactin	pseudomallei MSHR346	+237	+80	+338	+296	+706	+268	+398	-191	-562(+59)	+3300	+1739	0	+517	+737	+279	0
malleobactin	pseudomallei MSHR511	+237	+80	+338	+296	+706	+268	+398	-191	-562	+3288	+1739	0	+468	+737	+279	0
malleobactin	pseudomallei MSHR520	+237	+80	+340	+296	+706	+268	+398	-191	-562	+3293,6 (pseudo)	+1737,3 (pseudo)	0	+468	+737	+279	0
malleobactin	pseudomallei NAU20B-16	+237	+80	+338	+296	+706	+268	+398	-191	-562	+1828	+1739	0	+468	+737	+279	0
malleobactin	pseudomallei NCTC 13178	+237	+80	+338	+296	+706	+268	+398	-191	-562	+3295	+1739	0	+468	+737	+279	0
malleobactin	pseudomallei NCTC 13179	+237	+80	+338	+296	+706	+268	+398	-191	-562	+3300	+1738	0	+468	+736	+279	0
malleobactin	thailandensis E264-1	+237	+82	+363	+302	+659	+264	+419	-191	-562	+3296	+1772	0	+504	+738	+248	0
malleobactin	thailandensis E444	+237	+82	+338	+302	+706	+264	+397	-191	-562	+3296	+1763	0	+478	+738	+279	0
malleobactin	thailandensis H0587	+237	+82	+338	+305	+706	+264	+397	-191	-562	+3305	+1763	0	+478	+738	+279	0
malleobactin	thailandensis MSMB121	+230	+80	+338	+306	+710	+268	+354	0	-562	+3326	+1702	0	+473	+739	+279	0
malleobactin	xenovorans LB400	+223	+105	+340	+288	+704	+272	+276	0	-562	+3180	+1675	0	+456	-727	+280	0
											phm A	*phm* B					
phymabactin	phymatum STM815	+244	+77	0	+281	+708	+264	+341	0	-597	+3224	+1659	+341	+462	0	0	0
		orb S	*orb* H	*orb* G	*orb* C	*orb* D	*orb* F	orbB		*orb* E	*orb* I	*orb* J	*orb* K	*pvdA*	*orb* A	*pvd* F	*orb* L
ornibactin	ambifaria AMMD	+222	+80	+339	+ 283	+ 697	+266	+345	0	- 581	+ 3227	+ 1670	- 341	+ 458	+ 750	+279	+338
ornibactin	ambifaria MC40-6	+222	+80	+339	+283	+ 697	+266	+345	0	- 581	+ 3227	+ 1669	+ 341	+ 458	+ 753	+279	+338
ornibactin	cenocepia AU 1054	+222	+80	+335	+282	+696	+266	+341	0	-607	+3231	+1662	+343	+458	+754	+279	+338
ornibactin	cenocepia HI2424	+222	80	+335	+282	+696	+266	+341	0	-607	+3231	+1662	+343	+458	+754	+279	+338
ornibactin	cenocepia J2315	+222	+80	+335	+268	+696	+266	+297	0	-581	+3222	+1669	+345	+458	+755	+279	+340
ornibactin	cenocepia MC0-3	+222	+80	+335	+282	+696	+266	+341	0	-607	+3221	+1665	+343	+458	+754	+279	+338
ornibactin	cepacia GC4	+222	+80	+339	+290	+697	+266	+344	0	-581	+3232	+1661	+341	+458	+765	+279	+338
ornibactin	lata ASM1294v1	+218	+80	+335	+282	+696	+267	+345	0	-581	+3219	+1663	+344	+458	+750	+279	+338
ornibactin	multivorans ATCC 17616-1	+215	+80	+336	+280	+696	+266	+344	0	-581	+3219	+1658	+338	+454	+734	+279	+336
ornibactin	multivorans ATCC 17616-2	+213	+80	+336	+280	+696	+266	+344	0	-581	+3219	+1658	+338	+454	+739	+279	+336
ornibactin	sp. KJ006	+221	+80	0	283	+703	+269	+349	0	-579	+3259	+1661	0	+437	+757	+279	+336
ornibactin	vietnamiensis G4	+221	+80	+339	+283	+703	+269	+349	0	-579	+3242	+1661	0	+459	+773	+279	+336
ornibactin	sp. RPE64	221	99	+334	+278	+699	+259	+283	+43?	-583	+3181	+1655	0	+454	+750	+281	+319
ornibactin	sp. YI23	0	0	0	0	0	0	0	0	-582	+3167	1651	0	+453	+728	+277	+303

(colour key)	NRPS biosynthesis
(colour key)	Accessory enzymes
(colour key)	Uptake
(colour key)	Regulation
(colour key)	Unknown function
