# Supplementary material for: Burkholderia genome mining for nonribosomal peptide synthetases reveals a great potential for novel siderophores and lipopeptides synthesis
Source: Microbiologyopen. 2016 Apr 5;5(3):512–26. doi: 10.1002/mbo3.347 (PMC4906002; doi:10.1002/mbo3.347)
Supplement: Supplementary file 6 — Table S3. NRPSs of Burkholderia rhizoxinica HKI 454. A, adenylation domain; LCL, condensation between two l‐monomers; DCL, condensation between d‐monomer and l‐monomer; C/E, dual condensation domain catalyzing both epimerization and condensation; T, thiolation domain; E, epimerization domain; Te, Thioesterase domain; “?”, nonidentified domain; LP, lipopeptide. [file MBO3-5-512-s006.pdf]

Table S3

Cluster	putative product	gene	genetic support	NCBI annotation	strand	protein length (AA)	Protein-ID	domain architecture
1	LP7	RBRH_01504	plasmid pBRH01	non-ribosomal peptide synthetase module	plus	7658	YP_004021982.1	C_{start}-A-T-${}^{L}C_{L}$-A-T-${}^{L}C_{L}$-A-T-C/E-A-T-C/E-A-T-${}^{L}C_{L}$-A-T-C/E-A-T-Te
2	LP6	RBRH_00429	plasmid pBRH01	non-ribosomal peptide synthetase module	plus	6184	YP_004022028.1	A-T-${}^{L}C_{L}$-A-T-${}^{L}C_{L}$-A-T-${}^{L}C_{L}$-A-T-${}^{L}C_{L}$-A-T-C/E-A-T-Te
3	LP3	RBRH_00451	plasmid pBRH01	non-ribosomal peptide synthetase module	plus	181	YP_004022050	$C_{starter}$
		RBRH_00452	plasmid pBRH01	non-ribosomal peptide synthetase module	plus	2773	YP_004022052.1	A-T-${}^{L}C_{L}$-A-T-C/E-A-T-Te
4	LP4	RBRH_00484	plasmid pBRH01	non-ribosomal peptide synthetase module	plus	4512	YP_004022085.1	$C_{starter}$-A-T-C/E-A-T-${}^{L}C_{L}$-A-T-C/E-A-T-Te
5	LP3	RBRH_00572	plasmid pBRH01	non-ribosomal peptide synthetase module	minus	227	YP_004022168.1	T
		RBRH_00574	plasmid pBRH01	peptide synthetase	minus	799	YP_004022169.1	?-A
		RBRH_00575	plasmid pBRH01	Carveol dehydrogenase	minus	274	YP_004022170.1	KR
		RBRH_00576	plasmid pBRH01	thioesterase	minus	237	YP_004022171.1	Te
		RBRH_00578	plasmid pBRH01	non-ribosomal peptide synthetase module	plus	2515	YP_004022173.1	$C_{starter}$-A-T-${}^{L}C_{L}$-A-T-Te
6	LP3	RBRH_00622	plasmid pBRH01	non-ribosomal peptide synthetase module	plus	2505	YP_004022223.1	$C_{starter}$-A-T-C/E-A-T-${}^{L}C_{L}$
		RBRH_04248	plasmid pBRH01	non-ribosomal peptide synthetase module	plus	394	YP_004022224.1	A
		RBRH_00623	plasmid pBRH01	non-ribosomal peptide synthetase module	plus	402	YP_004022225.1	T
7	LP2	RBRH_00274	plasmid pBRH01	non-ribosomal peptide synthetase module	plus	2537	YP_004022375.1	?-A-T-C/E-A-T-Te
8	LP3	RBRH_00260	plasmid pBRH01	non-ribosomal peptide synthetase module	minus	3431	YP_004022385.1	$C_{starter}$-A-T-${}^{L}C_{L}$-A-T-C/E-A-T-Te
9	LP6	RBRH_01792	plasmid pBRH01	non-ribosomal peptide synthetase module	minus	6591	YP_004022432.1	$C_{starter}$-A-T-${}^{L}C_{L}$-A-T-${}^{L}C_{L}$-A-T-${}^{L}C_{L}$-A-T-${}^{L}C_{L}$-A-T-${}^{L}C_{L}$-A-T-Te
10	LP8	RBRH_04314	plasmid pBRH01	non-ribosomal peptide synthetase module	minus	1407	YP_004022559.1	T-${}^{L}C_{L}$-A-T-Te
		RBRH_02787	plasmid pBRH01	non-ribosomal peptide synthetase module	minus	4500	YP_004022560.1	A-T-${}^{L}C_{L}$-A-M-T-${}^{L}C_{L}$-A-T-C/E-A-M
		RBRH_04279	plasmid pBRH01	non-ribosomal peptide synthetase module	minus	4100	YP_004022561.1	$C_{starter}$-A-M-T-C/E-A-T-${}^{L}C_{L}$-A-T-${}^{L}C_{L}$
11	LP	RBRH_02642	chromosome 1	Non-ribosomal peptide synthetase modules	plus	2133	YP_004029261.1	A-M-T-C/E
		RBRH_04173	chromosome 1	Non-ribosomal peptide synthetase modules	plus	872	YP_004029262.1	A-T-${}^{L}C_{L}$
12	LP8	RBRH_03984	chromosome 1	Non-ribosomal peptide synthetase modules	minus	3538	YP_004029595.1	T$^{-L}C_{L}$-A-T-C/E-A-T-${}^{L}C_{L}$-A-T-Te
		RBRH_04313	chromosome 1	Non-ribosomal peptide synthetase modules	minus	2340	YP_004029596.1	T-${}^{L}C_{L}$-A-T-C/E-A
		RBRH_03867	chromosome 1	Non-ribosomal peptide synthetase modules	minus	2865	YP_004029597.1	$C_{starter}$-A-T$^{-L}C_{L}$-A-T-C
		RBRH_03865	chromosome 1	hypothetical protein	plus	118	YP_004029598.1	T
		RBRH_03864	chromosome 1	D-alanine-activating enzyme	plus	534	YP_004029599.1	A
13	LP10	RBRH_01506	chromosome 1	Non-ribosomal peptide synthetase modules	plus	5791	YP_004029720.1	A-T-C/E-A-T-${}^{L}C_{L}$-A-M-T-${}^{L}C_{L}$-A-T-${}^{L}C_{L}$-A-T-Te
		RBRH_04125	chromosome 1	Non-ribosomal peptide synthetase modules	minus	1086	YP_004029722.1	A-T-Te
		RBRH_00209	chromosome 1	Non-ribosomal peptide synthetase modules	minus	4454	YP_004029723.1	$C_{starter}$-A-T-C/E-A-T-${}^{L}C_{L}$-A-T-${}^{L}C_{L}$-A-T
