# Supplementary material for: Burkholderia genome mining for nonribosomal peptide synthetases reveals a great potential for novel siderophores and lipopeptides synthesis
Source: Microbiologyopen. 2016 Apr 5;5(3):512–26. doi: 10.1002/mbo3.347 (PMC4906002; doi:10.1002/mbo3.347)
Supplement: Supplementary file 7 — Protocol S1. Construction of a B. ambifaria AMMD mutant deleted in burkholdin (Bk) synthesis. The supplementary file describes the construction of a mutant strain deleted in the gene bksG (Bamb_6472) to support the relationship between the cluster bks and the production of an antifungal compound belonging to Bk family. [file MBO3-5-512-s007.pdf]

Protocol S1- Construction of a burkholdin deleted mutant of *B. ambifaria* AMMD

To identify the cluster dedicated to antimicrobial activity, we constructed a burkholdin-deficient mutant, *B.ambifaria* AMMD-Δbamb_6472, by deleting a 9,530-bp fragment within burkholdins–encoding gene *bamb_6472*.

1- Construction of pMQ30-Δ6472

The allelic replacement vector, PMQ30 (Shanks *et al*., 2006) was used for mutant construction. Two regions of the NRPS gene *bamb_6472* were amplified using primers Up6472-F and Up6472-R (1015 bp amplicon) and primers Down6472-F and Down6472-R (887 bp amplicon), respectively. These primers contained recombination sites for the plasmid. The PCR mix consisted of 25 µl of PCR Master Mix (Thermo Scientific Fermentas), 10 µl Q-solution (Qiagen), 1.25 µl of each primer (each at 20 µM), 7.5 µl water and 5 µl of genomic DNA. The reaction mixture was subjected to the following thermal cycles: one cycle at 94°C for 3min; 30 cycles (94°C, 30 s; 60°C, 45 s; 72°C, 2 min) and a final extension at 72°C for 10 min. Plasmid pMQ30 was purified using GeneJet plasmid miniprep kit (Thermo Scientific Fermentas). A 5 µl sample was checked on a 1% agarose gel. Subsequently, the purified plasmid was digested by a mix of 9.5 µl milliQ water, 4 µl Tango yellow buffer (2x), 5 µl plasmid pMQ30, 1 µl *Bam*HI and 0.5 µl *EcoR*I. The mix was incubated for 2h at 37°C. 5 µl of the plasmid digest was checked on 1% agarose and the concentration of the digest was also measured.

2- *In vivo* cloning in *S. cerevisiae* InvSc1

The PCR products of *bamb_6472* were cloned flanking each other through *in vivo* homologous recombination in the yeast, *S. cerevisiae* InvSc1, grown overnight in yeast peptone dextrose (YPD) at 30°C. Then 0.5 ml were centrifuged for 1 min at 3000 rpm. The cell pellet was washed with 0.5 ml lazy bones solution and the following was added to the mix: 20 µl of carrier DNA (2mg/ml), 45 µl of each PCR products UP and DOWN, 5 µl digested pMQ30 plasmid. The mix was homogeneized for 1 min on a Vortex and incubated overnight at room temperature. The mixture was subjected to heat shock for 12 min at 42°C. Then, cells were centrifuged for 1 min at 3000 rpm, washed with 0.6 ml TE buffer and the resulting pellet was further redissolved in 0.6 ml of TE

buffer and the cells were plated on SD- uracil medium. After 2 to 3 days of incubation, colonies were activated with 6 ml of SD-uracil medium. Further, plasmid was isolated using miniprep kit (Fermentas) and confirmed by PCR using primers Up6472-F and Down6472-R. 10 µl of the eluted plasmid were checked on 1% agarose gel.

3- Construction of AMMD- deficient mutant

Plasmid pMQ30-Δ6472 was introduced into *E. coli* WM3064 by electroporation. 2 µl of pMQ30 Δ6472 was added to 50 µl of thawed *E. coli* WM3064 competent cells. Mix was transferred to an electroporation cuvette and electroporated at 2.5 kV/200 ohms/25 µF. Electroporated cells were transferred into 1 ml of LB containing 2% of glucose and incubated in a rotary shaker for 1 h at 37°C. The cells were plated on LB containing 20 µg/ ml of gentamycin sulfate and 100mg/L of DAB (Diaminopimelic acid). Colony PCR was conducted using primers Up6472-F and Down6472-R to confirm the success of electroporation.

The plasmid was mobilized into *B. ambifaria* AMMD by conjugation. The donor strain *E.coli* WM3064+pMQ30-Δ6472 was grown in LB broth containing Gm 20 µg/ml and DAB 100mg/L. The acceptor strain, AMMD, was grown without antibiotics. The cells were centrifuged for 2 min at 5000 rpm, the supernatant was discarded and the cells were washed with 1. 5 ml LB. For conjugation, 200 µl of acceptor strain was mixed with 200 µl of donor strain. This mix was spotted on LB plates containing 100 mg/L DAB, dried, then plates were incubated overnight at 37 °C. Cell mass were then collected and suspended in 1 ml LB. For each conjugate, dilutions up to 10^{-3} were performed, plated on LB containing gentamycin 300 µg/ml and incubated overnight at 37 °C. Colonies were streaked on new LB plates with gentamycin 300 µg/ml. Colony PCR was performed to confirm the presence of the plasmid in merodiploid strain (cell containing plasmid) using primers Up6472-F and Down6472-R. One colony was cultured on LB and incubated overnight at 37 °C in a rotary shaker. Culture was diluted up to 10^{-5} and 100 µl were plated on LB containing 10% sucrose. Selected colonies were streak out on new LB plates without gentamycin.

Figure S2-1: Knock-out mutant for burkholdins/ occidiofungin production in *B. ambifaria* AMMD

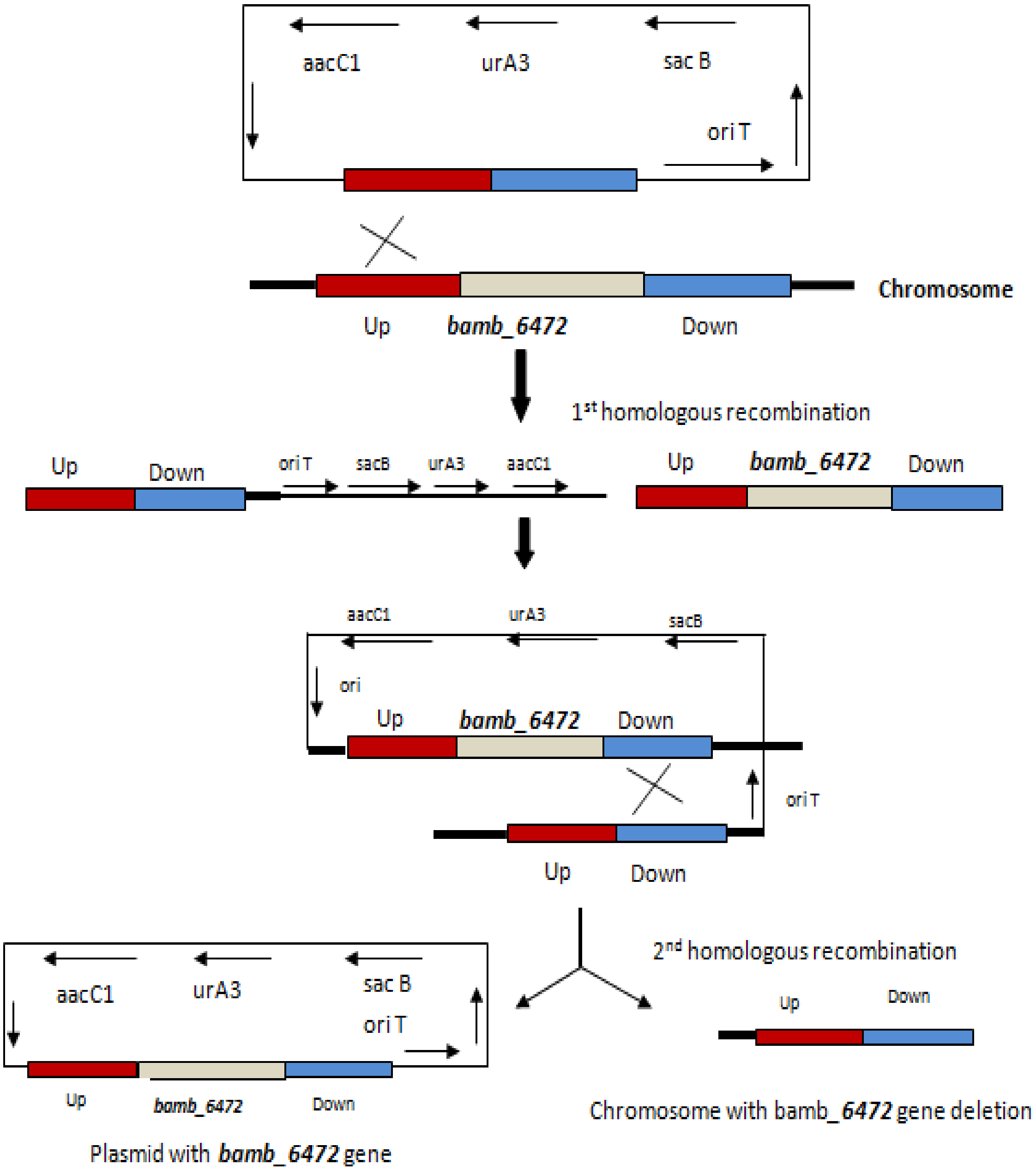

Colony PCR of these colonies was carried out using primers Up6472-F and Down6472-R to confirm and detect deleted mutants Fig. S5-2.

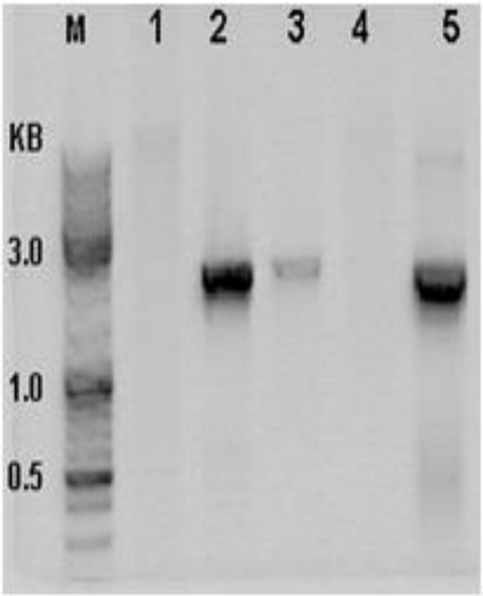

Fig. S2-2: Colony PCR analysis to confirm the deletion of bamb_6472 gene in the second recombination.

Lane M: 10 kb O'geneRuler (Thermo Fisher Fermentas)
Lane 1: wild-type showing no amplification (Fragment too long)
Lanes 2-3 : colonies of AMMDΔbamb_6472 allowing the corresponding amplification of 2000 bp
Lane 4 : Negative PCR
Lane 5 : pMQ30-Δbamb_6472 in recombination with bamb_6472 up and down.

Fig. S2-3 : MALDI-ToF detection of burkholdin produced by *B. ambifaria* AMMD

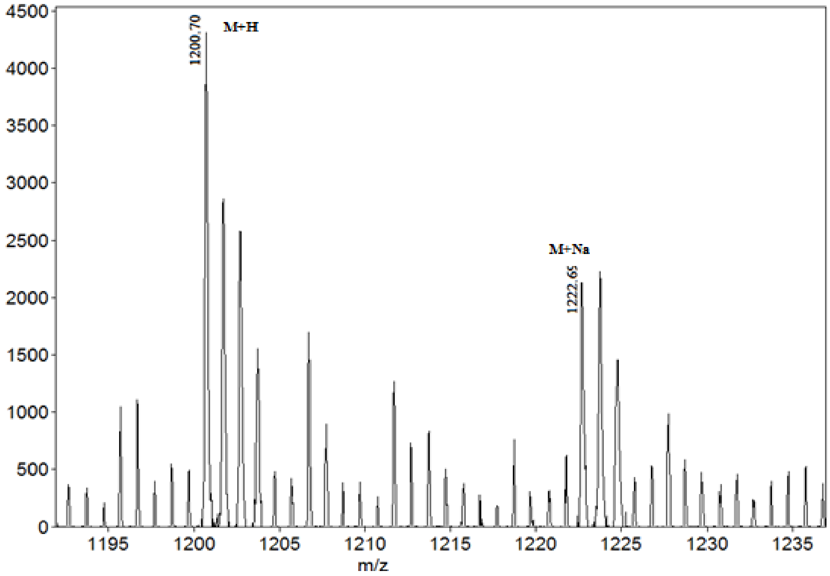

Shanks, R.M.Q., Caiazza, N.C., Hinsa, S.M., Toutain, C.M. and O'Toole, G.A. (2006) *Saccharomyces cerevisiae*-based molecular tool kit for manipulation of genes from gram-negative bacteria. *Appl. Environm. Microbiol.* **72**, 5027-5036.

Saltikov, C.W. and Newman, D.K. (2003) Genetic identification of a respiratory arsenate reductase. *Proc. Natl. Acad. Sci.* **16**, 10983-10988.
